# Mesenchymal stem cell therapy promotes the improvement and recovery of renal function in a preclinical model

**DOI:** 10.1590/1678-4685-GMB-2015-0178

**Published:** 2016-06-03

**Authors:** Antônio Urt-Filho, Rodrigo Juliano Oliveira, Larissa Correa Hermeto, João Renato Pesarini, Natan de David, Wilson de Barros Cantero, Gustavo Falcão, Guido Marks, Andréia Conceição Milan Brochado Antoniolli-Silva

**Affiliations:** 1 Centro de Estudos em Células Tronco, Terapia Celular e Genética Toxicológica, Hospital Universitário "Maria Aparecida Pedrossian", Empresa Brasileira de Serviços Hospitalares, Campo Grande, MS, Brazil; 2 Programa de Pós-Graduação em Saúde e Desenvolvimento na Região Centro-Oeste, Faculdade de Medicina "Dr. Hélio Mandetta", Universidade Federal de Mato Grosso do Sul, Campo Grande, MS, Brazil; 3 Programa de Mestrado em Farmácia, Centro de Ciências Biológicas e da Saúde, Universidade Federal de Mato Grosso do Sul, Campo Grande, MS, Brazil; 4 Programa de Pós-Graduação em Clínica Veterinária, Faculdade de Ciências Agrária e Veterinária, Universidade Estadual Paulista "Júlio de Mesquita Filho", Jaboticabal, SP, Brazil; 5 Faculdade de Medicina "Dr. Hélio Mandetta", Universidade Federal de Mato Grosso do Sul, Campo Grande, MS, Brazil

**Keywords:** bone marrow, chemotherapy, nephrotoxicity, kidney disease, tissue regeneration

## Abstract

Acute renal failure (ARF) is an extremely important public health issue in need of novel therapies. The present study aimed to evaluate the capacity of mesenchymal stem cell (MSC) therapy to promote the improvement and recovery of renal function in a preclinical model. Wistar rats were used as the experimental model, and our results show that cisplatin (5mg/kg) can efficiently induce ARF, as measured by changes in biochemical (urea and creatinine) and histological parameters. MSC therapy performed 24h after the administration of chemotherapy resulted in normalized plasma urea and creatinine levels 30 and 45d after the onset of kidney disease. Furthermore, MSC therapy significantly reduced histological changes (intratubular cast formation in protein overload nephropathy and tubular hydropic degeneration) in this ARF model. Thus, considering that current therapies for ARF are merely palliative and that MSC therapy can promote the improvement and recovery of renal function in this model system, we suggest that innovative/alternative therapies involving MSCs should be considered for clinical studies in humans to treat ARF.

## Introduction

Acute renal failure (ARF) is characterized by the accumulation of nitrogen-containing compounds (urea and creatinine) in the blood (with or without oliguria) followed by the loss of renal function, ultimately blocking the excretion of waste nitrogen and maintenance of homeostasis (Thadhani *et al.*, 1996).

ARF is associated with high mortality and morbidity rates and is reported in approximately 4-5% of all hospitalization cases. This disease can develop into more severe forms, resulting in death in approximately 50-70% of patients, making ARF an extremely important public health issue (Hoste and Schurgers, 2008; Wen *et al.*, 2010).

ARF can be caused by a number of factors, including decreased renal perfusion without cellular injury, an ischemic, toxic or obstructive insult to the renal tubules, a tubule-interstitial process with inflammation and edema, or a primary reduction of glomerular filtering capacity, among others (Thadhani *et al.*, 1996). Chemo- and radiotherapy are also important causes of ARF (Humphreys *et al.*, 2005). Among chemotherapeutic drugs, cisplatin, which is used to treat the majority of solid and hematological tumors (Serpeloni *et al.*, 2013), is most associated with nephrotoxicity due to its high concentrations in the kidneys, even at non-toxic plasma levels, as well as its adverse impact on the renal transport system (Gordon and Gattone 1986; Ren *et al.*, 2002; Serpeloni *et al.*, 2013).

Generally, ARF is incurable, and current therapeutic strategies involve simply removing or treating its cause (Fry and Farrington, 2006). As ARF is prone to complications and chronicity, innovative/alternative therapies for the quick, safe and efficient recovery of renal function are increasingly required.

Currently, models of ARF involving cell therapy have been effective and promising (Yokoo *et al.*, 2003; Brodie and Humes, 2005; Zerbini *et al.*, 2006 Choi *et al.*, 2010; Papazova *et al.*, 2015). In the present study, we investigated the capacity of mesenchymal stem cell (MSC) therapy to promote the improvement and recovery of renal function in a pre-clinical model.

## Material and Methods

### Experiment 1

#### Chemical agents

ARF was induced by means of a single-dose administration of the chemotherapeutic agent cisplatin (Fauldcispla LIBBS®, Brazil, Lot: 12GO702) at 5mg/kg body weight (bw) via intraperitoneal (ip) injection.

#### Animals

Fifty-nine sexually mature male Wistar rats were obtained from the Central Animal House of the Center of Biological and Health Sciences of the Federal University of Mato Grosso do Sul (Centro de Ciências Biológicas e da Saúde da Universidade Federal de Mato Grosso do Sul – CCBS/UFMS). The animals were housed in groups of three in polypropylene cages containing wood shavings as bedding and were fed a commercial diet (Nuvital®, Brazil) and filtered water *ad libitum*. The cages were kept in a ventilated cabinet (Alesco®, Brazil) under standard climate-controlled conditions with a 12-h photoperiod (12:12 h LD), a temperature of 222 °C, and a relative humidity of 5510%. The experiment was conducted in accordance with the guidelines of the Universal Declaration of Animal Rights and was approved by the Animal Research Ethics Committee of the UFMS (Protocol #499/2012).

#### Experimental design

Initially, the animals were divided into two lots. Lot I consisted of 10 animals, which were used as bone marrow-derived MSC donors. Lot II consisted of 49 animals, which were further subdivided into three experimental groups. Control Group (CG; *n* = 14) animals were treated with a single dose of 1 mL/100g (bw; ip) phosphate-buffered saline (PBS), followed by an intravenous (iv) dose of 1 mL PBS via tail vein injection after 24 h. Control animals were evaluated at 30 days (CG-30d; *n* = 7) and 45 days (CG-45d; *n* = 7) after the onset of treatment. The ARF Group (ARFG; *n* = 21) animals were treated with a single dose of cisplatin at 5 mg/kg (bw; ip), followed by 1 mL PBS (iv) 24-h later. ARF animals were then evaluated at 48 h (ARFG-48h; *n* = 7), 30 days (ARFG-30d; *n* = 7), and 45 days (ARFG-45d; *n* = 7) after cisplatin treatment. The ARFG + MSC Group (ARFG+MSC; *n* = 14) animals were treated with a single dose of cisplatin at 5mg/kg (bw; ip), followed by treatment with MSCs in 1 mL PBS (iv) 24h later. Animals were then evaluated at 30days (ARF+MSC-30d; *n* = 7) and 45days (ARFG+MSC-45d; *n*=7) after cisplatin treatment.

#### Assessment of renal function and confirmation of acute renal failure

ARF was confirmed indirectly by measuring serum urea and creatinine levels. Peripheral blood was collected under anesthesia (ketamine – 50 mg/kg bw, ip; xylazine – 10mg/kg bw, ip) from the retro-orbital plexus, either at 48h in the ARFG group (used as the standard for the establishment of ARF), or at 30d and 45d after cisplatin administration in the other groups. The first blood sample was drawn from animals in the ARFG-48h group 7d prior to the administration of cisplatin, which was used as an internal control for the experiment. After collection, the blood was allowed to sediment at 4 °C, and the serum was then stored at −20 °C until the time of analysis. Analyses were performed using an automated COBAS 6000 analyzer (Roche Diagnostics ®, Brazil), according to the manufacturer's recommendations.

#### Isolation and expansion of bone marrow-derived mesenchymal stem cells

Donor animals from Lot I were euthanized by anesthetic overdose (Thiopentax® Cristalia Laboratories, Brazil, Lot: 12075226). Femurs were then collected under sterile conditions, and the bone marrow was flushed with 5mL PBS supplemented with 1% antibiotics (penicillin/streptomycin, LGB Biotecnologia®, Brazil). Cell suspensions were centrifuged for 5 min at 670 x g, and the pellet was resuspended with 5 mL sterile PBS; resuspension was performed three times. The pellet was then resuspended with 4 mL Dulbecco's Low Glucose Modified Eagle's Medium (DMEM-low glucose; LGC Biotecnologia®, Brazil), transferred to a conical tube containing 4 mL SeptCell (LGC Biotecnologia®, Brazil), and then centrifuged for 30 min at 241 x g. The mononuclear cell fraction was collected and resuspended with 5 mL PBS, centrifuged for 5 min at 670 x g, and then resuspended with PBS. This last step was repeated five times before the pellet from each donor animal was seeded into two 25-cm^2^ culture flasks containing 10 mL of DMEM-low glucose supplemented with 10% Fetal Calf Serum (LGC Biotecnologia®, Brazil) and 1% antibiotics (penicillin/streptomycin, LGB Biotecnologia, Brazil). Cell viability was assessed by adding an equal volume of trypan blue (LGC Biotecnologia®, Brazil) to 10 μL samples from each donor.

Cells were cultured in a CO_2_ incubator (Thermo Scientific®, USA) at 37 °C under 5% CO_2_. During expansion, the supplemented culture medium was replaced every 48 h. During each passage (cultures at 70-80% confluence), the cells were washed three times with 5 mL PBS and then trypsinized by adding 1 or 2 mL of 0.25% trypsin (LGC Biotecnologia®, Brazil) to the 25- or 75-cm^2^ flasks, respectively. The trypsin was inactivated by adding 5 volumes of supplemented culture medium. The cell suspension was then centrifuged for 5 min at 670 x g, and the pellet was resuspended with 1 mL of supplemented culture medium. For the first passage, each 25-cm^2^ flask was divided into two 25-cm^2^ flasks. For the second and third passages, cells were seeded into 75-cm^2^ flasks at 1:1 and 1:2 ratios, respectively. During each passage, 10 μL samples were collected for trypan blue analysis of cell viability.

#### Osteogenic, adipogenic and chondrogenic differentiation

Flasks intended for differentiation experiments were trypsinized as described above after reaching 80% confluence; four 25-cm^2^ flasks were seeded with 2 10^5^ cells each, and two 15-mL conical tubes were seeded with 1 10^6^ cells each. Two 25-cm^2^ flasks and one conical tube were used as controls and cultured with supplemented culture medium. Cells used for osteogenic and adipogenic differentiation were preincubated with supplemented culture medium for 24 h and then cultured for 14 d with culture medium from the STEMPRO Osteogenesis or Adipogenesis Differentiation Kit (Invitrogen®, USA), respectively. For chondrogenic differentiation, cells were pre-incubated in supplemented medium for 2 h and then cultured for 21 d with culture medium from the STEMPRO Chondrogenesis Differentiation Kit (Invitrogen®, USA). Culture media from all of the above differentiation cultures were changed twice weekly. At the end of the respective culture periods, media were removed and cells were fixed in paraformaldehyde for 1 h. Adipogenic and osteogenic differentiation were assessed by staining with Oil Red O and Alizarin Red S, respectively. Chondrogenic differentiation was assessed by submitting the pellet to conventional histological processing and staining with toluidine blue (Hermeto *et al.*, 2015).

#### Mesenchymal stem cell transplantation

After reaching confluence during the third passage, 49 culture flasks originated from the first unique 10 donors were trypsinized and randomly transplanted to the 49 receptor animals from the different experimental groups. Suspended cells were centrifuged for 5 min at 670 x g, and the pellet was resuspended with PBS to obtain 1.0 × 10^6^ cells for use in cell therapy. Animals from the ARFG+MSC received MSC transplant (iv) 24 h after cisplatin administration, whereas animals from the remaining groups were treated with 1mL of MSC suspension vehicle (sterile PBS; iv). For transplantation, the animals were anesthetized as described above.

#### Histopathological analysis

At the end of each experimental period (48 h, 30 d and 45 d), the animals were euthanized by an anesthetic overdose, as described above. Kidneys were collected, sectioned, fixed in 10% buffered formaldehyde and prepared according to routine histopathological practices. Briefly, tissue fragments were dehydrated after fixation, cleaned, and then embedded in paraffin. Samples were prepared using a microtome (4-μm thick slices) and stained with hematoxylin/eosin (HE) for histopathological analysis by bright-field microscopy (1000x magnification).

Slides were submitted to double-blind histopathological analysis according to the Banff 97 Classification Criteria: 1) tubulitis: intratubular lymphocyte infiltration (necrosis and the degree of fibrosis were not evaluated); 2) tubular hydropic degeneration: cytoplasmic ballooning of cells in the proximal convoluted tubule (1+ < 25%; 2+ 25–75%; 3+ > 75%); 3) intratubular cast formation: eosinophilic deposits within tubules (proximal convoluted and distal tubule, Henle's loop, failure of glomerular filtration, failure of tubule reabsorption) (1+ < 25%; 2+ 25–75%; 3+>75%); 4) glomerulitis: lymphocyte infiltration in the glomeruli; 5) arteritis: lymphocyte infiltration in the arterioles; 6) interstitial infiltration: lymphocytes in the interstitial space (1+ 0–25%; 2+ 25–75%; 3+ > 75%); 7) interstitial fibrosis: scar collagen deposition; 8) glomerulosclerosis: fibrosis in the glomeruli (nephron failure); 9) calcifications; 10) cortical retraction/atrophy: partial substitution of kidney parenchyma by fibrosis and/or inflammatory infiltration; 11) tubular apoptosis/necrosis: total or partial tubule degeneration (1+ < 25%; 2+ 25–75%; 3+>75%; and 12) global necrosis: involvement of the glomeruli and tubules (Racusen *et al.*, 1999).

#### Statistical Analysis

Statistical analysis was performed using the SigmaPlot statistics software program (Version 12.5), with the significance level set at 5%. The applied non-parametric tests included the Mann-Whitney *U* test for comparisons between two experimental groups, and the Kruskal-Wallis test, followed by Dunn's test, for comparisons between more than two experimental groups.

### Experiment 2: Mesenchymal stem cell localization and migration

#### Experimental animals, design and conditions

Six sexually mature male Wistar rats were divided into two experimental groups (*n* = 4): ARFG and ARFG+MSC. Animals were evaluated 48 h after the transplantation of MSCs that were previously stained with the nuclear marker 4’,6-diamidino-2-phenylindole (DAPI, Life Technologies®). Animal housing conditions, determination of renal function, confirmation of ARF, anesthesia, and euthanasia, as well as isolation, expansion and transplantation of MSCs were performed as described for Experiment 1. However, prior to transplantation, MSCs were incubated with 10 μg/mL of DAPI in the dark for 30 min at 37 °C.

#### Histological fluorescence analysis

After euthanasia, animal kidneys were collected and immediately frozen in liquid nitrogen. Organs were then mounted on a holder with cryostat embedding medium (EasyPath), and 5-μm thick slices were cut at −10 °C in the dark. Sections were placed on slides and analyzed by fluorescence microscopy (BA410 FL) with a DAPI filter (EX D350/50x; DM 400DCLP; BA D460/50m) at 400x magnification.

## Results

### Expansion of mesenchymal stem cells

After the isolation procedure, MSCs were subjected to an expansion protocol with consecutive passages. For all passages, only cultures with > 95% viability were maintained.

### Mesenchymal stem cells differentiate into osteogenic, adipogenic and chondrogenic cells in vitro

**Figure 1:**
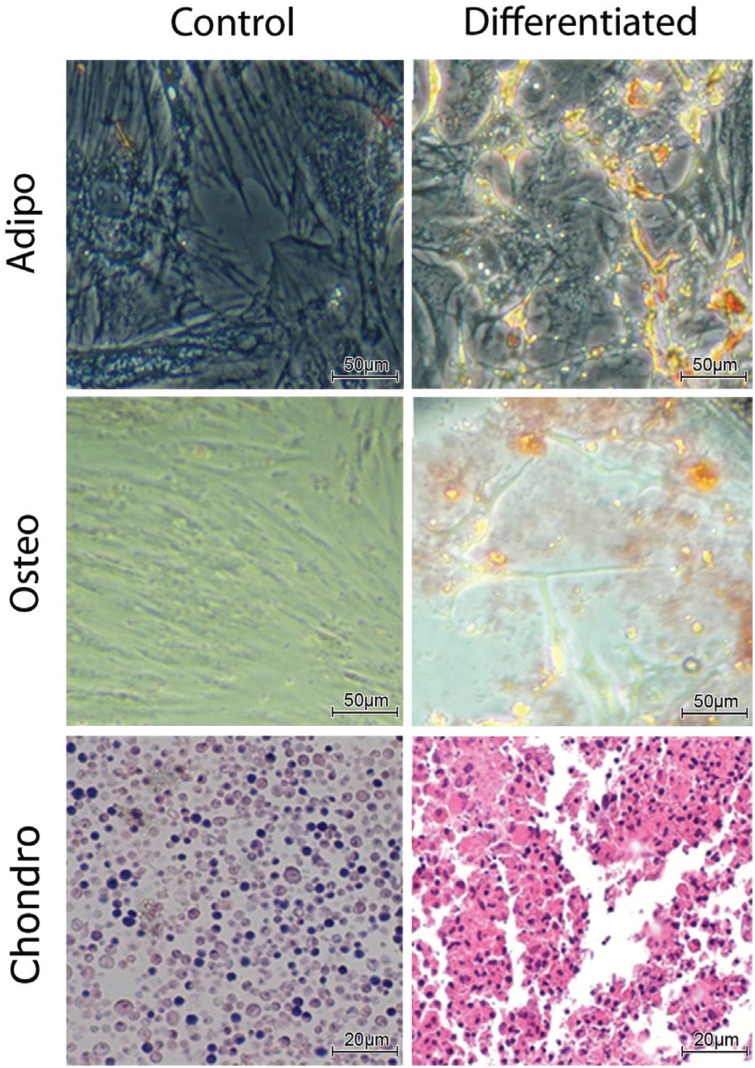
MSC are able to differentiate into adipogenic, osteogenic (scale bars: 50 μm) and chondrogenic (scale bars: 20 μm) lineages, as shown by the staining.

Bone-marrow-derived MSCs obtained from donor rats were induced to differentiate, and characteristic morphological changes were observed in these cell types. Figure 1 shows representative images of undifferentiated control cells cultured on an adherent surface (Control), as well as adipogenic and osteogenic differentiation (Differentiation). Osteogenesis was assessed by means of Alizarin Red S staining, which labels calcium deposits in differentiated cells, and adipogenesis was assessed by means of Oil Red O staining, which labels fat vesicles and vacuoles in adipogenic cells. The lower row shows undifferentiated cells from pellet cultures (Control), and chondrogenic differentiated MSCs, the latter showing toluidine blue staining, indicating increased extracellular matrix deposition by differentiated chondrocytes.

### Mesenchymal stem cell localization and migration

**Figure 2:**
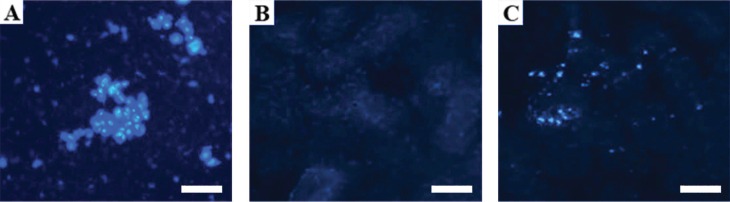
DAPI staining: (A) MSC stained with DAPI; (B) renal cortex without MSC infiltration; (C) renal cortex with MSC infiltration, 48 h after the transplant. Scale bars: 20 μm.

DAPI-stained MSCs migrated from the bloodstream to the kidneys, and their localization was confirmed by fluorescence microscopy. Figure 2 shows a stained MSC suspension (A), a renal cortex with no evidence of MSC infiltration (B), and a renal cortex exhibiting MSCs stained with DAPI (C).

### Validation of the cisplatin-induced acute renal failure model

**Figure 3:**
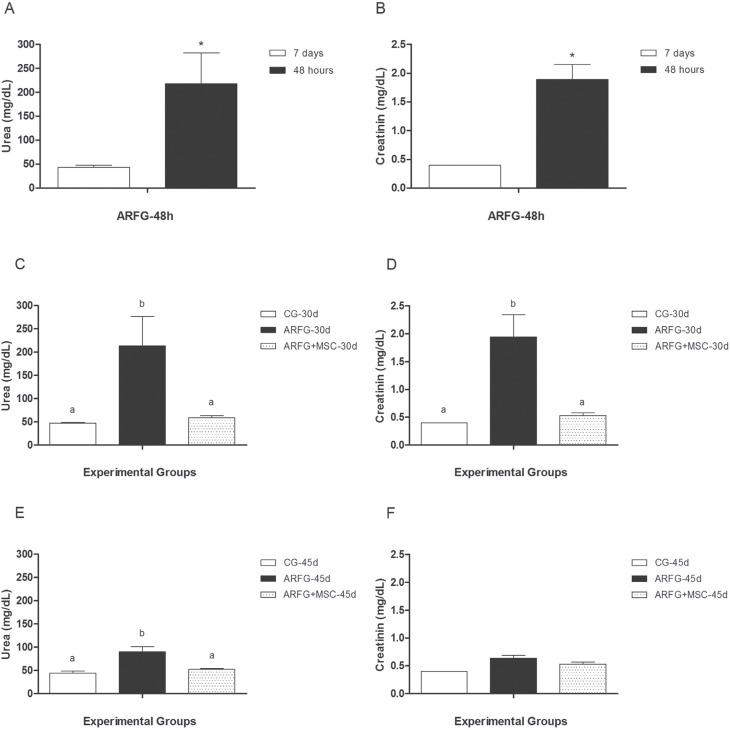
Urea and creatinine plasmatic concentration (mean ± SD). A and B) Confirmation of acute renal failure. ARFG-48h: Acute renal failure group; animals were treated with cisplatin, evaluated seven days before and 48 h after the administration of the chemotherapy. C and D) Animals were evaluated 30 days after the administration of the chemotherapy. E and F) Animals were evaluated 45 days after the administration of the chemotherapy. CG: Control group. ARFG: Acute renal failure group; animals treated with cisplatin. ARFG+MSC: In this group, MSC were transplanted to animals with acute renal failure 24 h after the chemotherapy administration. Statistical analysis: A and B: Mann-Whitney, *statistically significant differences, p £ 0,05; C to F: Kruskal-Wallis with Dunn's post-hoc test; different letters indicate statistically significant differences, p ≤ 0.05.

Plasma creatinine and urea levels were measured in the ARFG-48h group 7 d before and 48 h after the administration of cisplatin, and statistically significant increases in the levels of these biomarkers confirmed the onset of ARF (Figure 3A,B). Mean urea and creatinine levels were 43.43 ± 4.07 and 0.40 ±0.00, respectively; after cisplatin administration, levels increased (p≤0.05) to 218.00 ± 64.42 and 1.89 ±0.26, respectively.

Based on urea and creatinine levels 30 d after the administration of chemotherapy in the ARFG group, we concluded that cisplatin treatment successfully induced ARF, which persisted throughout the experimental period, exhibiting mean values of 213.29 ±63.23 and 1.94 ±0.40, respectively. By contrast, in the ARFG+MSC, transplantation of MSCs 24h after chemotherapy led to improved renal function, with mean urea and creatinine values of 59.14 ± 4.53 and 0.53 ±0.05, respectively. Importantly, these values were statistically similar (p > 0.05) to those from the CG group (47.43 ±1.54 and 0.40 ±0.00 for urea and creatinine, respectively).

At 45 d, creatinine levels were no longer significantly different (p > 0.05) between experimental groups, whereas urea levels remained high (p ≤ 0.05) in the ARFG group (90.5 ±10.56) compared with the CG (44.14 ±4.36) and ARFG+MSC (52.57 ±1.19) groups; the latter two groups were not significantly different from one another (p > 0.05).

### Histopathological analysis

Histopathological analysis of the ARFG-48h group revealed tubular hydropic degeneration, intratubular cast formation, tubular necrosis/apoptosis, and global necrosis, with medians of 3, 3, 2 and 1, respectively.

**Figure 4:**
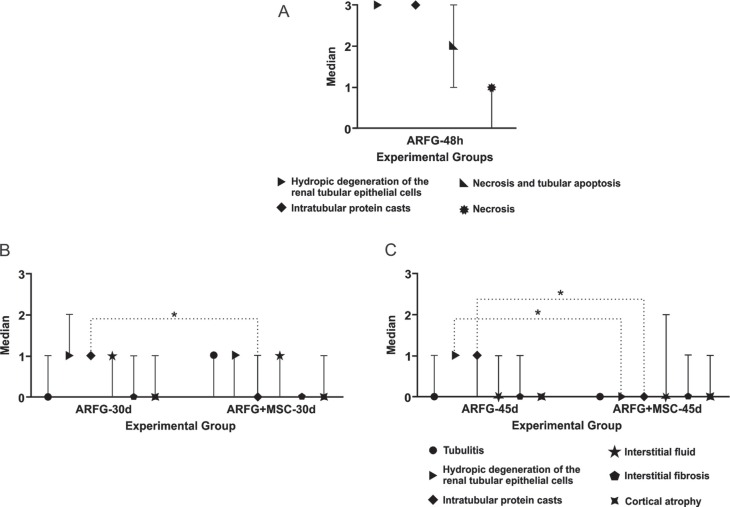
Data from the histopathological cuts according the Banff 97 classification. A) ARFG-48h: Acute renal failure group; animals were treated with cisplatin, evaluated seven days before and 48 h after the administration of the chemotherapy. B and C) ARFG: Acute renal failure group; animals treated with cisplatin. ARFG+MSC: In this group, MSC were transplanted to animals with acute renal failure 24 h after the chemotherapy administration. B) 30d – animals evaluated after 30 days of the administration of the chemotherapy. C) 45d – animals evaluated after 45 days of the administration of the chemotherapy. Statistical analysis: Mann-Whitney, *statistically significant differences, p ≤ 0.05.

**Figure 5:**
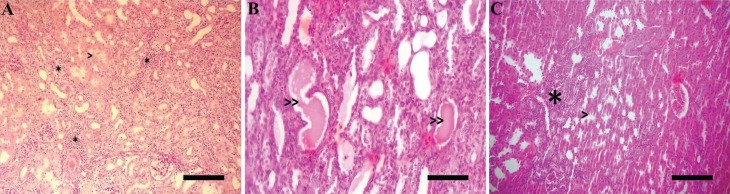
Histological sections of the kidneys from cisplatin treated and MSC transplanted animals. A) Hydropic degeneration (>) and interstitial infiltration (*); scale bar: 100 μm. B) Intratubular cast formation (>>); scale bar: 25 mm. C) interstitial fibrosis (>) and interstitial infiltration (*); scale bar: 100μm.

In the ARFG and ARFG+MSC groups, the observed changes were tubulitis, tubular hydropic degeneration, intratubular cast formation, interstitial infiltration, interstitial fibrosis, and cortical atrophy – irrespective of the period of analysis (30 d or 45 d) (Figure 4A, Figure 5).

Comparisons between the ARFG-30d and the ARFG+MSC-30d groups revealed a statistically significant reduction in intratubular cast formation, with medians of 1 and 0, respectively. Conversely, there was no statistically significant difference between the ARFG-30d and the ARFG+MSC-30d groups with respect to tubulitis, tubular hydropic degeneration, interstitial infiltration, interstitial fibrosis, or cortical atrophy (medians of 0, 1, 1, 0 and 0 *vs.* 1, 1, 1, 0 and 1, respectively) (Figure 4B).

When comparing the ARFG-45d and the ARFG+MSC-45d groups, there was a statistically significant reduction in tubular hydropic degeneration (medians of 1 and 0, respectively) and intratubular cast formation (medians of 1 and 0, respectively). No significant differences were observed between the ARFG-45d and ARFG+MSC-45d groups with respect to tubulitis, interstitial infiltration, interstitial fibrosis, or cortical atrophy (medians of 0, 1, 1, 0 *vs.* 0, 0, 0, 0, respectively) (Figure 4C).

## Discussion

Preclinical models are currently being used to assess the effects of MSCs on various nephropathies. Of the several types of kidney disease that affect humans, renal failure is the primary target of modern bioengineering approaches, as cell therapy can replace specific cells types that have been lost in renal patients (Brodie and Humes, 2005). Such studies have addressed how cell therapy can help regenerate convoluted tubules and promote the normalization of biochemical parameters after an ischemic or toxic insult to the kidney (Lin *et al.*, 2003; Lin, 2006). However, the mechanisms involved in these processes remain poorly understood, and further studies are needed to extend these findings.

The MSCs used in the present study were characterized by adipogenic, osteogenic and chondrogenic differentiation. These results are consistent with previous studies (Bagnaninchi *et al.*, 2011; Pendleton *et al.*, 2013; Vieira *et al.*, 2014; Hermeto *et al.*, 2015), as was the efficiency of the selection and expansion methods used here.

The migration of MSCs to the kidney and the permanence of these cells remain controversial topics. However, in the present study, MSCs were stained with DAPI prior to intravenous transplantation, and they were identified in the kidney 48h after cell therapy. This result is consistent with other studies showing the presence of MSCs in the kidney (Ross *et al.*, 2012; Tang *et al.*, 2012; Pan *et al.*, 2014). In addition to nuclear staining with DAPI, other protocols have also been used to detect MSCs in the kidneys, including tagging with green fluorescent protein (Qiu *et al.*, 2014), detection of β-galactosidase (β-Gal) (Klinkhammer *et al.*, 2014), and transplantation of cells derived from different sexes (*e.g.*, male donor and female recipient) (Grimm *et al.*, 2001). Despite these studies, some authors have reported an absence of MSCs in the kidney in experiments performed with MSCs and ARF (Duffield *et al.*, 2005).

These discrepancies aside, studies involving MSC transplants have yielded good results (Altun *et al.*, 2012; Liu *et al.*, 2012; Xinaris *et al.*, 2013; Erpicum *et al.*, 2014), and in general, exogenous MSC donor sources are used (Morigi *et al.*, 2004), as was the case in the present study.

Our results demonstrate that ARF is efficiently induced by treatment with cisplatin – a widely used cancer therapy drug whose major complication is nephrotoxicity (Nishihara *et al.*, 2013; Hagar *et al.*, 2015). Urea and creatinine levels were used as biomarkers to confirm ARF induction, and these were increased 48h after chemotherapy administration. ARF was also confirmed histologically by detecting tubular hydropic degeneration, intratubular cast formation, tubular necrosis and apoptosis, and global necrosis. We also observed that these biochemical and histological changes persisted 30d after cisplatin administration. At 45 d, only increased urea levels and histological damage could still be observed, whereas creatinine levels were back to normal (*i.e.*, not significantly different from the control group). These observations suggest that a single dose of 5 mg/kg (bw; ip) cisplatin in Wistar rats is sufficient to efficiently induce ARF by toxic insult in this experiment model. Thus, this model can be used in studies to evaluate the events associated with patients under chemotherapy during the occurrence of ARF.

With respect to MSC transplantation, this method proved to be effective at improving the overall health of the animals undergoing cell therapy. Specifically, plasma urea and creatinine levels were efficiently reduced to levels similar to those found in the control group 30 d after the administration of chemotherapy. Additionally, histological analysis revealed significantly reduced intratubular cast formation over the same time period.

Forty-five days after cisplatin administration, the transplanted MSCs were still able to reduce plasma urea levels, yielding values similar to those in the control group, and histological analysis revealed statistically significant reductions in both tubular hydropic degeneration and intratubular cast formation.

The above results are consistent with other studies showing reductions in urea and creatinine levels in animals with ARF following cell therapy (Shih *et al.*, 2013; Geng *et al.*, 2014; Jiang *et al.*, 2015). Furthermore, in the studies by Imberti *et al.* (2007) and Qiu *et al.* (2014), improvements in histological parameters were also observed in animals with ARF who received MSC transplants.

Considering these results, we conclude that cell therapy with MSCs has therapeutic potential for ARF. Unlike some other organs, such as the heart or brain, the kidneys possess a high regenerative capacity following ischemic or toxic insult, and tissue repair is thought to involve three phases: (I) inflammation – recruitment of immune cells; (II) regeneration – substitution of injured cells with new cells of the same type; and (III) repair – healing of interstitial tissues (Bonventre, 2003; Lin *et al.*, 2008). More specifically, tubules regenerate to recover renal function through elongation, mitosis and differentiation of the remaining uninjured cells, which ultimately line the denuded basement membrane (Thadhani *et al.*, 1996; Sheridan and Bonventre, 2000; Devarajan, 2006; Humphreys *et al.*, 2008).

With respect to the capacity of MSCs to promote renal tissue repair, these cells are thought to act by: (I) modulating the inflammatory response, which can induce phagocytosis in the spleen or liver to remove the MSCs from the bloodstream, where they are present for a short time (Duffield and Bonventre, 2005; Duffield *et al.*, 2005); (II) secreting growth factors; (III) merging with injured cells; or (IV) differentiating into new resident renal cells (Duffield and Bonventre, 2005; Tögel *et al.*, 2005; Humphreys and Bonventre, 2008).

Our results support the notion that the effects of cell therapy are not based on repopulation and/or fusion of MSCs with the renal tubule (Duffield and Bonventre, 2005; Tögel *et al.*, 2005; Humphreys and Bonventre, 2008). However, in the present study, MSCs were found in the kidney 48h after cell therapy, and according to Morigi *et al.* (2006), Humphreys and Bonventre (2007, 2008), this time period is insufficient for the repopulation, fusion and/or transdifferentiation of MSCs towards reconstituting the renal epithelium. Also, the absence of MSCs on the renal tissue, observed by DAPI staining on days 5, 15, 30 and 45 (data not shown), corroborate these facts. An alternative explanation for the observed results is modulation at the paracrine level, considering that MSC therapy is related to increases in vascular endothelial growth factor (VEGF), hepatocyte growth factor (HGF) and insulin-like growth factor (IGF) 1 (Lange *et al.*, 2005; Tögel *et al.*, 2005), as well as with reduced inflammation, through the induction of anti-inflammatory cytokines (Krampera *et al.*, 2006). Another possibility is that MSCs trigger renal stem cells to undergo mitosis, differentiate, and migrate, thus promoting renal tissue regeneration. Indeed, the latter hypothesis appears to be the most widely accepted mode of action (Oliver, 2004; Oliver *et al.*, 2004; Lin *et al.*, 2005).

In view of the above, the present study is original because it demonstrated that the therapy with MSCs could be an alternative for patients under chemotherapy treatment who developed ARF. Also, considering the fact that cancer is a growing public health concern and the majority of treatments involve aggressive chemical components, this kind of therapy could be an important alternative to improve the health quality of patients. Thus, considering that current therapies for ARF are merely palliative and that MSC therapy promotes the recovery of renal function in this model, we suggest that innovative/alternative therapies involving MSCs should be considered for clinical studies in humans to treat ARF.
